# Ocean acidification impairs embryonic development and disrupts ink sac gene expression in the pharaoh cuttlefish (*Sepia pharaonis*)

**DOI:** 10.3389/fphys.2026.1979863

**Published:** 2026-10-23

**Authors:** Shaogang Li, Jiawen Liang, Jiahe Liu, Yongsen Lv, Junhao Peng, Ming Zeng, Chunyan Guo, Haiyang Zhang, Jigui Yuan, Liyun Wang

**Affiliations:** 1 College of Life Science and Technology, Lingnan Normal University, Zhanjiang, China; 2 College of Food Science and Technology, Guangdong Ocean University, Zhanjiang, China; 3 College of Marine Science, Beibu Gulf University, Qinzhou, China

**Keywords:** gene expression, ink sac genes, ocean acidification, pH conditions, *Sepia pharaonis*

## Abstract

**Background:**

Ocean acidification (OA) represents a growing threat to marine biodiversity, particularly affecting the sensitive early life stages of invertebrates. While the physiological impacts of OA are widely recognized, its specific effects on organogenesis and the molecular regulation of defensive traits in cephalopods remain poorly understood. This study investigated the influence of acidified seawater on the embryonic development and ink sac formation in the Pharaoh cuttlefish (*Sepia pharaonis*).

**Methods:**

Embryos were reared under four pH conditions (8.0, 7.7, 7.3, and 6.5). Embryonic growth, survival, hatching success, and ink sac morphogenesis were monitored throughout development. The expression of melanin synthesis–related genes, including *tyr, per*, and *spNOS*, was quantified using qRT-PCR.

**Results:**

Ocean acidification impaired embryonic development in a pH-dependent manner. Severe acidification (pH 6.5) resulted in complete mortality and no surviving embryos by Day 9, while moderate acidification (pH 7.3) led to delayed development and failure to hatch by Day 11, compared to the hatching observed in the control group. Ink sac development was markedly inhibited under acidified conditions, resulting in reduced size and pigmentation. Consistent with these morphological abnormalities, the expression of melanin synthesis markers, including tyrosinase (*tyr*) and peroxidase (*per*), exhibited a biphasic response, with early transient induction followed by consistent downregulation across all acidified treatments, while, the expression of the regulatory gene *Sepia pharaonis* nitric oxide synthase (*spNOS*) was significantly suppressed under severe acidification.

**Conclusions:**

These findings provide evidence that OA is associated with disrupted embryogenesis and impaired ink sac formation in *S. pharaonis*, accompanied by altered expression of melanin synthesis-related genes. The resulting reduction in defensive capacity may increase the vulnerability of hatchlings and potentially affect population recruitment under future ocean acidification scenarios.

## 1 Introduction

The Pharaoh cuttlefish (*Sepia pharaonis*) is an ecologically and economically important cephalopod species widely distributed across the Indo-Pacific region (Farhadi and Anderson, 2021; Song W. et al., 2021). In recent years, advances in artificial breeding and aquaculture have enabled large-scale cultivation of this species, providing a stable source for scientific and industrial use. Beyond its commercial value, *S. pharaonis* has drawn considerable attention for its unique biological features, particularly its ink sac, a specialized organ that plays a dual role in defense and biomedicine (Jiang et al., 2019). As a defensive mechanism, the ink sac enables the rapid release of melanin-rich ink, which confuses predators and enhances survival in the wild. In traditional and modern medicine, cuttlefish ink and other body components have been recognized for their pharmacological properties. Classical Chinese texts such as The Divine Farmer’s Materia Medica and the Compendium of Materia Medica describe cuttlefish ink as possessing hemostatic, analgesic, and astringent effects. Modern pharmacological studies have confirmed some of these functions, revealing that compounds derived from cuttlefish ink can induce apoptosis in cancer cells, inhibit tumor formation, and exhibit antimicrobial and preservative activities (Hernandez-Zazueta et al., 2021; Xiao et al., 2025). These findings highlight the ink sac as a key adaptation for predator evasion but also as a valuable source of marine bioactive substances with potential applications in medicine and biotechnology.

The proper functioning of the ink sac depends on its normal development and the biosynthesis of melanin, the primary pigment responsible for ink coloration. Melanin synthesis is tightly regulated by key enzymes such as tyrosinase (*tyr*) and peroxidase (*per*), which catalyze successive steps in the conversion of tyrosine into eumelanin (Tief et al., 1996). Tyrosinase acts as the rate-limiting enzyme, catalyzing the hydroxylation of tyrosine to L-DOPA and its subsequent oxidation to dopaquinone (Nagatsu et al., 2022), while peroxidase contributes to the polymerization of downstream intermediates such as DHI and DHICA (Snyman et al., 2024). In addition to these core enzymes, upstream regulatory molecules, including nitric oxide synthase (NOS) and N-methyl-D-aspartate (NMDA) receptors, modulate melanin synthesis by influencing tyrosinase activity through nitric oxide and cGMP signaling pathways (Cabaco et al., 2022). Previous studies in related species, such as *Sepia esculenta* and *Sepia officinalis*, have demonstrated that NOS is expressed in ink gland cells and participates in the regulation of tyrosinase-mediated melanogenesis (Palumbo, 2003; Scheinker et al., 2005). Collectively, these findings establish a clear molecular framework for understanding ink sac formation and provide the theoretical basis for examining how environmental stressors may interfere with this developmental process.

Among these stressors, ocean acidification (OA) has emerged as a major global concern. Driven by the continuous absorption of anthropogenic carbon dioxide (CO_2_) by the ocean, OA results in increased hydrogen ion (H^+^) concentration, reduced carbonate ion (CO_3_^2-^) availability, and a decline in seawater pH (Hofmann et al., 2019). Since the onset of the Industrial Revolution, atmospheric CO_2_ levels have risen dramatically, and oceanic pH has already dropped by approximately 0.1 units, a trend projected to continue over the coming centuries (Muller and Gruber, 2024). OA is known to exert broad physiological and ecological impacts on marine organisms, particularly those with high metabolic rates or limited buffering capacity. Cephalopods, including squid, octopus, and cuttlefish, possess a relatively strong capacity for active acid–base regulation during the adult stage, which can partially buffer external pH fluctuations (Birk et al., 2018). However, this regulatory capacity is not fully developed during early life stages. Embryos, characterized by limited ion-regulatory machinery, undeveloped physiological systems, and constrained energy reserves, are therefore considerably more vulnerable to environmental acidification. As a result, cephalopod embryogenesis represents a critical window of heightened sensitivity to ocean acidification (Gutowska et al., 2010; Hu et al., 2015). The energetic cost of maintaining ionic balance through branchial ion exchange can divert energy from growth and development, while pH-dependent reductions in hemocyanin oxygen-binding affinity may further compromise metabolism. Experimental studies on some cuttlefish species have shown that low-pH/elevated-CO_2_ conditions can reduce hatching success, impair growth and metabolic performance, and alter behavior, such as increased stress responses or changes in shelter-seeking and hunting (Moura et al., 2019). These findings indicate that OA may disrupt physiological homeostasis and defense-related behaviors in cephalopods, yet its potential influence on earlier developmental stages remains poorly understood.

Despite growing awareness of the ecological consequences of ocean acidification, little is known about its effects on cephalopod embryogenesis, particularly on the morphogenesis and molecular regulation of specialized organs such as the ink sac. The embryonic stage represents a critical window of vulnerability, during which organogenesis and molecular differentiation are highly sensitive to environmental perturbations. Given the pivotal role of the ink sac in both survival and medicinal value, understanding how acidified conditions affect its development is essential for assessing the broader implications of ocean change. Based on the established roles of *tyr*, *per*, and *NOS* in melanin synthesis and ink sac regulation, we hypothesize that ocean acidification impairs ink sac formation in *S. pharaonis* embryos by downregulating key genes involved in melanin biosynthesis.

To test this hypothesis, we simulated both projected ocean acidification scenarios and extreme acidification conditions relevant to coastal environments (pH 7.7, 7.3, and 6.5, with pH 8.0 as the control) and examined their effects on embryonic development, ink sac morphology, and the expression of melanin synthesis–related genes using quantitative real-time PCR (qRT-PCR). This study aims to elucidate the molecular mechanisms underlying the developmental sensitivity of cuttlefish embryos to ocean acidification. The findings are expected to enhance our understanding of cephalopod developmental biology under changing oceanic conditions and to inform the conservation and sustainable utilization of marine medicinal bioresources in an increasingly acidified world.

## 2 Materials and methods

### 2.1 Experimental materials

Live *Sepia pharaonis* egg masses were collected from the coastal waters of Zhanjiang, Guangdong Province, China, in April 2021 and transported to the Aquatic Animal Laboratory at Lingnan Normal University for incubation and experimental use. Prior to experimentation, the embryos were maintained under controlled laboratory conditions with continuous aeration and stable temperature regulation.

The experimental seawater was filtered through a 0.45 μm microporous membrane through a filtration system (Model APS-F, Sunsun Group Co., Ltd., Zhejiang, China) before use. For the preparation of acidified seawater, the filtered seawater was equilibrated with a high-purity CO_2_/air mixture by an aerator (Model DHG-1.5, Shandong Zhangqiu Blower Co., Ltd., Shandong, China) for 1–2 h in temperature-controlled conditions. The pH was continuously monitored and precisely adjusted to the target levels (7.70, 7.30, or 6.50) solely by regulating the flow rate of the CO_2/_air mixture. No chemical acids (such as acetic acid or HCl) were used for pH adjustment. The prepared seawater was then sealed until use. Control seawater was aerated without CO_2_ enrichment (pH 8.00-8.10). Subsequently, pH and dissolved oxygen (DO) were measured using a pH meter (Mettler Toledo DELTA320) to confirm that the target pH levels (7.70, 7.30, or 6.50) were achieved and stable. Seawater pH was verified daily using an independent portable pH meter (Mettler Toledo DELTA320 pH meter), which was calibrated with NIST-traceable standard buffers prior to each measurement. All treatments met strict water quality standards, maintaining a pH fluctuation within ±0.1 units and DO saturation above 90% throughout the experimental period.

### 2.2 Cuttlefish egg culture, observation, and sampling

Prior to the experiment, *Sepia pharaonis* embryos were pre-cultured for three days under controlled laboratory conditions (temperature: 28 ± 1 °C; salinity: 23 ± 1‰; total alkalinity: 2.1 ± 0.1 meq/L) to ensure uniform developmental stages. Egg clutches were collected from 15 different females, and eggs from each clutch were randomly and evenly distributed across the four treatment groups to ensure that each group contained offspring from all parental sources. Subsequently, the eggs were randomly distributed into four seawater treatment systems: three acidified treatments (pH 7.70, 7.30, and 6.50) and one control (pH 8.00). Each treatment consisted of three replicate tanks (n = 3), totaling 12 tanks. Each experimental tank had a total volume of 1.5 m³ with an effective water volume of 1.2 m³. Embryos were suspended on nylon ropes within perforated plastic baskets supported by foam blocks, with a stocking density of 30 to 50 embryos per tank (approximately 25 to 42 embryos per m³ of effective water volume. A semi-static culture system was employed, with continuous aeration (24 h/day) and mechanical filtration to maintain water quality.

Throughout the experiment, temperature, salinity, DO, and pH were monitored daily, while total alkalinity (TA) was measured using a Hanna HI755 Alkalinity Photometer (HANNA Instruments, Shanghai, China). Additional carbonate system parameters, including the partial pressure of CO_2_ (*p*CO_2_) and aragonite saturation state (Ω-arag), were calculated from the measured variables using the CO2SYS software (version 2.1). The mean (± SD) values of all measured and calculated water parameters for each treatment are summarized in **Table 1**.

**Table 1:** Seawater carbonate system parameters for the four experimental pH treatments.

Parameter	pH8.0 (control)	pH 7.7	pH 7.3	pH 6.5
Salinity (‰)	23 ± 1	25 ± 2	26 ± 1	25 ± 2
Total Alkalinity (meq/L)	2.1 ± 0.1	2.1 ± 0.1	2.1 ± 0.1	2.1 ± 0.1
Temperature (°C)	28 ± 1	28 ± 1	28 ± 1	28 ± 1
Dissolved Oxygen Saturation (%)	98 ± 1	98 ± 1	98 ± 1	98 ± 1
*p*CO_2_ (µatm)	415 ± 35	1350 ± 150	3150 ± 350	17500 ± 2500
Aragonite Saturation State (Ω-arag)	3.75 ± 0.25	1.75 ± 0.25	0.75 ± 0.15	0.15 ± 0.05

Values are presented as mean ± standard deviation. Temperature, salinity, pH, and total alkalinity (TA) were measured directly. The partial pressure of CO_2_ (*p*CO_2_) and the aragonite saturation state (Ω-arag) were calculated from measured pH, TA, temperature, and salinity using the CO2SYS software.

Each day, 15 embryos were randomly sampled from each treatment (5 embryos per tank × 3 tanks). Embryos within each tank were derived from a mixed pool of egg clutches originating from 15 different females, and were evenly and randomly distributed prior to incubation. Thus, daily sampling was performed independently within each tank to ensure random selection from a genetically mixed population. The embryos were dissected under a stereomicroscope (SE2200, Ningbo Yongxin Optical Co., Zhejiang, China) to examine organogenesis, including eye, arm, and ink sac development. The ink sac, eyes, and arms were carefully isolated, photographed using ScopeImage 9.0 software, and immediately frozen in liquid nitrogen. For RNA extraction, five ink sacs from each tank were pooled into a single biological replicate, and all samples were stored at -80 °C freezer (Qingdao Haier Biomedical Co., Ltd., Shandong, China).

In parallel, an additional five intact embryos from each tank were collected daily to assess developmental stages according to Chen et al (Dao-Hai et al., 2012). After removal of the outer egg capsule, embryos were observed and photographed under the stereomicroscope. Developmental stages were recorded, and samples were snap-frozen and stored at -80 °C. For subsequent analyses, sample groups were labeled according to the number of days post-exposure.

To quantitatively assess the effects of seawater acidification on embryonic development, several indices were measured daily. Water quality parameters were measured with a pH meter (Model PH828+, Dongguan Wanchuang Electronic Products Co., Ltd., Guangdong, China) and a seawater hydrometer (Model AR8212+, Dongguan Wanchuang Electronic Products Co., Ltd., Guangdong, China), Seawater alkalinity was determined using a microcomputer seawater alkalinity photometer (Model H1755, Hanna Instruments Co., Ltd., Shanghai, China).

Experimental observations were conducted using a stereomicroscope (Model SE2200, Ningbo Yongxin Optical Co., Ltd., Zhejiang, China). Mantle length and yolk sac area were recorded using calibrated imaging software to provide continuous, quantitative tracking of overall embryonic growth. Additionally, ink sac area, length, and width were measured each day to evaluate organ morphogenesis and pigmentation development.

### 2.3 Embryo viability assessment

Embryo viability was assessed daily based on morphological and behavioral criteria. Embryos were classified into three categories. Normal development, characterized by progressive organogenesis (including the formation of eyes, arms, and ink sac), continuous yolk sac absorption, and the presence of spontaneous movement. Developmental arrest, defined by cessation of organogenesis, absence of movement, and abnormal yolk sac appearance (e.g., turbidity, opacity, or hardening). Death, indicated by complete absence of movement, yolk disintegration, or visible tissue necrosis.

To confirm mortality, embryos were gently stimulated under a stereomicroscope using a fine dissection needle. Individuals that failed to exhibit any movement within 30 s of mechanical stimulation were classified as dead.

### 2.4 Experimental endpoints

The experimental endpoints varied among treatments based on developmental progression and survival status. For the control (pH 8.0) and pH 7.3 groups, the experiment was terminated on Day 11, when control embryos initiated hatching; the pH 7.3 group remained viable until Day 11 but exhibited marked developmental retardation and increased mortality. For the pH 6.5 and pH 7.7 groups, data collection was terminated on Day 9. The pH 6.5 group exhibited complete developmental arrest and mortality, while the pH 7.7 group showed pronounced developmental delay and failure to hatch.

### 2.5 Total RNA extraction and cDNA synthesis

Total RNA was extracted from ink sac tissues collected under the four pH treatments using TRIZOL A+ Reagent (TIANGEN, Beijing, China) according to the manufacturer’s protocol. Approximately 100 mg of frozen tissue was ground into a fine powder in liquid nitrogen using a pre-chilled mortar and pestle, then transferred to a 1.5 mL RNase-free centrifuge tube for RNA extraction.

RNA integrity was verified by 1% agarose gel electrophoresis. RNA purity and concentration were determined using a NanoDrop spectrophotometer (Thermo Scientific, Waltham, MA, USA). Only samples with an A260/A280 ratio between 1.8 and 2.0 and an A260/A230 ratio between 2.0 and 2.2 were used for subsequent cDNA synthesis.

First-strand cDNA was synthesized using the HiScript III RT SuperMix for qPCR (+gDNA wiper) (Vazyme, R223, Nanjing, China) following the manufacturer’s instructions. For each reaction, 50 ng–2 μg of total RNA was used in a final volume of 20 μL.

### 2.6 Primer design and qualitative PCR

Primers were designed based on homologous sequences obtained from previously published embryonic transcriptome databases of the closely related species *Sepiella japonica* (Accession Nos. SRP200876 and SRP170983) (Liyun et al., 2020; Wang et al., 2020). To ensure target specificity for *Sepia pharaonis*, the sequences were validated by alignment with orthologous sequences from *Sepia officinalis* available in GenBank. The ribosomal protein L23a (*rpl23a*) gene was selected as the internal reference gene. Target genes included Tyrosinase (*tyr*), Nitric Oxide Synthase (*spNOS*), and Peroxidase (*per*). The primer sequences and expected product sizes are listed in **Table 2**.

**Table 2:** Primer sequences used for quantitative real-time PCR (qRT-PCR).

Gene		5'--3'	Product (bp)
*tyr*	F	GTCGTTGTGCCTTACTGGGA	160
	R	AGTTTCGGTGCAAAGGTCCA	
*per*	F	AGAACCACCCCGACAGATGA	118
	R	TTGGCTGGACATTGACTCGG	
*spNOS*	F	CAATGGTCGAAGGGGACGAA	137
	R	GTGTAGGGCTCCTGTTGCTT	
*rpl23a*	F	CCGGGCCAAGAGAGCTATTC	112
	R	CTTAGGGTCACGAGGTTGCC	

The table lists the forward (F) and reverse (R) primer sequences for the target genes (*tyr*, *per*, *spNOS*) and the reference gene (*rpl23a*), along with their expected amplicon sizes in base pairs (bp).

The specificity of each primer pair was confirmed by melting curve analysis and agarose gel electrophoresis. Following each qPCR amplification, a melting curve was generated by gradually increasing the temperature from 65 °C to 95 °C while continuously monitoring fluorescence. All target genes displayed a single, sharp peak on the melting curve, indicating the absence of non-specific amplification or primer-dimer formation. To further validate product specificity, 2% agarose gel electrophoresis was performed, revealing single, bright bands of the expected sizes for all genes.

Amplification efficiency was determined by constructing standard curves using serial two-fold dilutions of high-quality cDNA (six dilution levels). The efficiency (E) of each primer pair was calculated from the slope of the standard curve using the equation: E=[10^(−1/Slope)^−1]×100%. All primer pairs exhibited amplification efficiencies between 90% and 110%, with correlation coefficients (R^2^) greater than 0.99, meeting the criteria for reliable qPCR quantification.

### 2.7 Quantitative real-time PCR

The relative expression levels of *tyr*, *spNOS*, and *per* in ink sac samples collected daily (Days 1-9) from each pH treatment were quantified using qRT-PCR. The *rpl23a* gene was used as the internal reference for normalization. Reactions were performed using the PowerUp™ SYBR Green Master Mix (Thermo Fisher Scientific, USA) on an ABI StepOnePlus system (Applied Biosystems, USA), following the manufacturer’s protocol.

Each 20 μL reaction mixture contained 10 μL of SYBR Green Master Mix, 0.4 μL of each primer (10 μM), 1 μL of cDNA template, and 8.2 μL of nuclease-free water. The PCR cycling parameters were as follows: Initial denaturation at 95 °C for 90 s; followed by 40 cycles of: Denaturation at 95 °C for 5 s, annealing at 60 °C for 15 s, extension at 72 °C for 20 s. A melting curve was subsequently generated from 65 °C to 95 °C to verify product specificity.

Each biological sample was analyzed in triplicate, with three technical replicates per biological replicate to ensure data reliability. No-template controls (NTCs) were included to detect potential contamination or primer-dimer formation.

Relative gene expression levels were calculated using the 2^-ΔΔCt^ method, with expression values normalized to *rpl23a* and compared among pH treatments and developmental days. The results were expressed as mean ± standard deviation (SD).

### 2.8 Reference gene stability validation

To validate the stability of *rpl23a* as an internal reference gene under our experimental conditions, we analyzed its raw Ct values across all samples (4 pH treatments × 9 developmental days × 3 biological replicates, n = 108). The coefficient of variation (CV) was calculated to assess expression stability. According to established guidelines (Hellemans et al., 2007), a CV below 10% indicates acceptable stability for a reference gene.

### 2.9 Data analysis

Each pH treatment consisted of three independent replicate tanks, which were treated as biological replicates for statistical analyses. Relative expression levels of the target genes were calculated using the 2^-ΔΔCT^ method with *rpl23a* as the internal reference gene and the control group (pH 8.0, Day 1) as the calibrator.

Statistical analyses were conducted using GraphPad Prism v10.2.1 (GraphPad Software, USA). Data were tested for normality and homogeneity of variance prior to analysis. To account for temporal dependency and avoid pseudoreplication, developmental and morphometric data were analyzed using Two-way ANOVA (factors: pH treatment, developmental time, and their interaction). When a significant interaction or main effect was detected, Tukey’s multiple-comparison test was performed to identify specific differences among pH treatments at each time point. To address the potential confounding effects of salinity, an Analysis of Covariance (ANCOVA) was performed with salinity as a covariate. If the covariate was not significant (p > 0.05), it was removed from the final models. Data are presented as mean ± standard deviation (SD), and statistical significance was defined as p < 0.05. The complete statistical results for all pairwise comparisons at each time point are provided in **Supplementary Tables S2–S4**.

## 3 Results and analysis

### 3.1 Ocean acidification reduces survival, delays development, and inhibits hatching

Embryonic development of *Sepia pharaonis* was monitored under four pH treatments (8.0, 7.7, 7.3, and 6.5), revealing pronounced pH-dependent effects on morphology, growth and viability (**Figure 1**; **Supplementary Figure S1**). On day 1, embryos in all treatments appeared morphologically similar, corresponding to approximately Stage 17 of development, characterized by a large, transparent yolk sac and a well-defined mantle (Dao-Hai et al., 2012). By Day 4, developmental divergence became apparent. Embryos in the control (pH 8.0) and moderate acidification groups (pH 7.7 and 7.3) had progressed normally, displaying mantle expression, yolk sac absorption, and the appearance of chromatophores. In contrast, embryos exposed to severe acidification (pH 6.5) exhibited clear developmental retardation, with no visible chromatophore formation and a persistently large yolk sac. These differences become more pronounced by Day 7. Control embryos showed advanced organogenesis and continued yolk utilization, whereas embryos at pH 7.7 and pH 7.3 exhibited slower developmental progression. Under severe acidification (pH 6.5), embryonic growth was markedly impaired, and the yolk sac become opaque, indicating developmental dysfunction. By Day 11, control embryos had completed development and hatched. Embryos reared at pH 7.3 remained viable until Day 11 but exhibited substantial developmental retardation and increased mortality. In contrast, embryos exposed to pH 7.7 showed pronounced developmental delay and failed to hatch, with data collection terminated on Day 9. In contrast, embryos exposed to pH 6.5 failed to complete organogenesis and displayed severe abnormalities, including yolk sac degeneration, tissue deterioration, and complete mortality.

**Figure 1:**
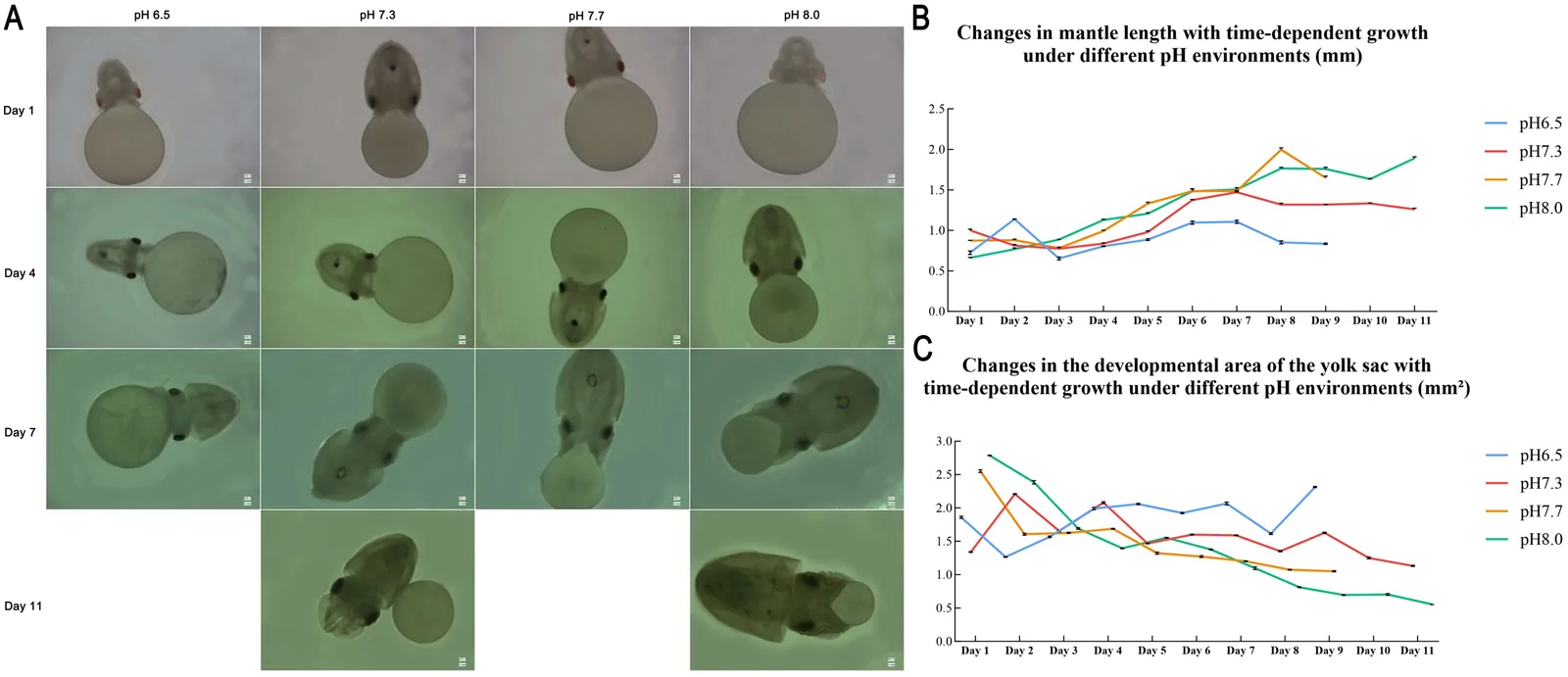
Effects of low pH on the embryonic development of *Sepia pharaonis*. **(A)** Representative morphological images of embryos from Day 1 to Day 9 under control (pH 8.0) and acidified (pH 7.7, 7.3, and 6.5) conditions. Developmental arrest and yolk opacification are evident in the pH 6.5 group from Day 6 onwards. Morphological observations for the pH 6.5 and pH 7.7 groups were terminated on Day 9 due to complete mortality and failure to hatch, respectively. **(B)** Time-dependent changes in mantle length (mm) across the four pH treatments. **(C)** Time-dependent changes in yolk sac area (mm²) across the four pH treatments, indicating yolk absorption rates. In both line graphs, blue, red, orange and green lines correspond to the pH 6.5, pH 7.3, pH 7.7 and pH 8.0 groups, respectively. Data points in **(B, C)** represent the mean ± SD (n = 3 replicate tanks, with 5 embryos per tank treated as subsamples). Statistical significance for all pairwise comparisons at each time point is provided in **Supplementary Table S2** (mantle length) and **Supplementary Table S3** (yolk sac area).

Morphometric measurements supported these observations. Mantle length increased continuously in the control group across the entire experimental period, while acidification exerted non-monotonic, pH-dependent effects on embryonic growth. Severe acidification (pH 6.5) strongly suppressed somatic growth, with embryos showing minimal increase in mantle length throughout the experiment. Under moderate acidification, the two groups followed distinct growth trajectories. Mantle length in the pH 7.7 group accelerated after Day 5 and transiently outpaced controls between Days 7 and 9, whereas the pH 7.3 group consistently displayed slower growth relative to the control (**Figure 1**; **Supplementary Figure S2**). Conversely, yolk sac area declined most rapidly in the control group, reflecting efficient nutrient utilization, whereas yolk absorption was markedly impaired under severe acidification.

Yolk sac area, an indicator of nutrient absorption, displayed an inverse trend to mantle length (**Figure 1**; **Supplementary Figure S3**). Embryos reared under control conditions (pH 8.0) showed the most rapid yolk sac absorption, consistent with normal developmental progression. Acidified treatments exhibited delayed yolk utilization in a pH-dependent manner. While embryos at pH 7.7 displayed a continuous reduction in yolk reserves throughout development, those at pH 7.3 retained relatively large yolk sacs until the late developmental stages. In contrast, embryos exposed to severe acidification (pH 6.5) showed minimal yolk sac absorption, with yolk reserves remaining largely intact throughout the experiment. This persistent yolk retention was accompanied by developmental arrest and severe morphological abnormalities, indicating a substantial impairment of embryonic growth and nutrient utilization under extreme acidification. Detailed statistical significance for mantle length and yolk sac area at each time point is summarized in **Supplementary Tables S2**, **S3**.

### 3.2 Ink sac formation is severely impaired by ocean acidification

Ink sac morphogenesis was examined throughout embryonic development under different pH conditions (**Figure 2**; **Supplementary Figure S4**). On Day 1, nascent ink sac primordia appeared as tiny pigmented dots across all four treatment groups. Acidified groups showed earlier and more distinct pigment deposition in the primordial ink sac, while the ink sac structure in the control group was fainter and less prominent at this early stage. By Day 4, distinct oval-shaped ink sacs had formed in all treatments except for the pH 6.5 group. By Day 7, substantial differences in ink sac development were evident. Control embryos displayed enlarged and pigmented ink sacs with a characteristic metallic sheen, indicative of active melanogenesis. Similar but less pronounced development was observed in the pH 7.7 and pH 7.3 groups. In contrast, embryos exposed to pH 6.5 exhibited small, pale, and poorly developed ink sacs that lacked reflective pigmentation.

**Figure 2:**
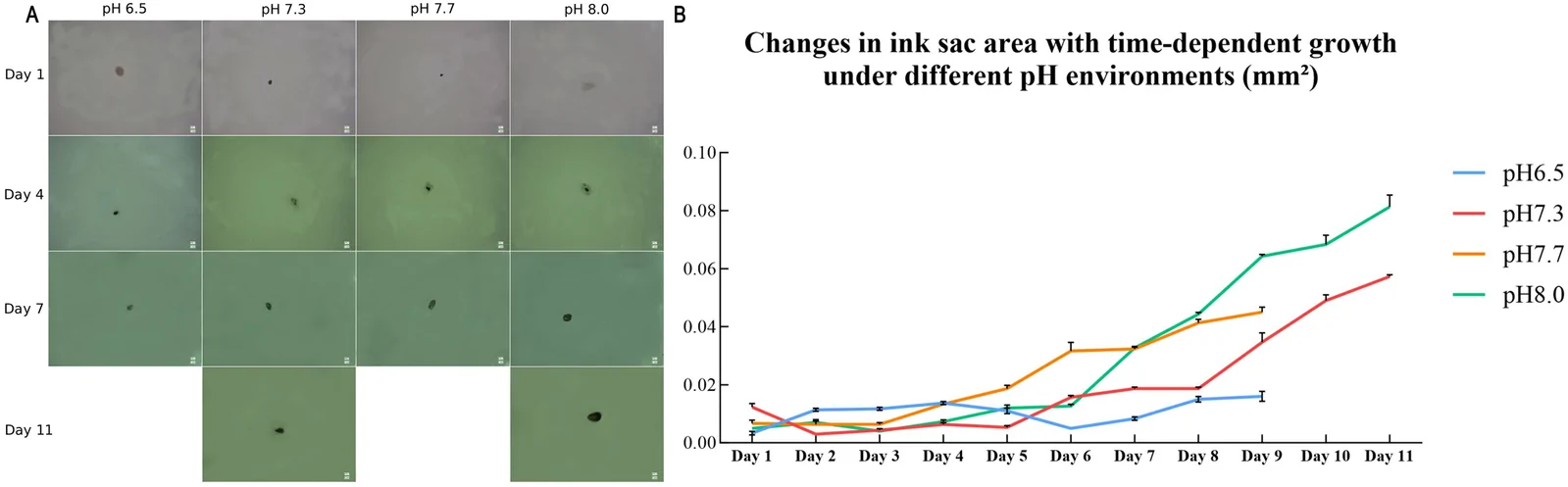
Effects of low pH on the morphogenesis of the ink sac in *Sepia pharaonis* embryos. **(A)** Representative images illustrating the development of the ink sac from Day 1 to Day 9. Note the lack of pigmentation and developmental arrest in both the pH 6.5 and pH 7.7 groups. Note: Morphological observations for the pH 6.5 and pH 7.7 groups were terminated on Day 9. **(B)** Quantitative analysis of the changes in ink sac area (mm²) over time across the four pH treatments. Data points represent the mean ± SD (n = 3 replicate tanks, with 5 embryos per tank treated as subsamples). Statistical significance for all pairwise comparisons at each time point is provided in **Supplementary Table S4**.

Quantitative analysis confirmed these morphological observations (**Figure 2**; **Supplementary Figure S5**). Ink sac area increased steadily throughout development in the control group but was progressively reduced under decreasing pH conditions. Embryos exposed to pH 7.3 exhibited limited expansion of the ink sac during late development, whereas those maintained at pH 6.5 showed only minimal growth followed by developmental stagnation. Overall, these results indicate that seawater acidification not only delays general embryonic development but also specifically disrupts ink sac morphogenesis and pigmentation. Detailed statistical significance for ink sac area at each time point is summarized in **Supplementary Table S4**.

### 3.3 Validation of *rpl23a* stability as an internal reference gene

To ensure the reliability of qPCR data, the expression stability of the candidate reference gene *rpl23a* was evaluated across all experimental conditions. Across all 108 samples, the Ct values of *rpl23a* ranged from 17.68 to 28.79, with a mean of 20.17 ± 1.67 (SD). The coefficient of variation (CV) was 8.27%, which is within the acceptable range for stable reference genes (< 10%) (Hellemans et al., 2007).

Analysis across different pH treatments revealed CV values of 6.81%, 7.60%, 7.37%, and 10.31% for pH 8.0, 7.7, 7.3, and 6.5, respectively. Across developmental days, CV values ranged from 5.38% (Day 1) to 12.07% (Day 9). Although a slight increase in variability was observed at Day 9 under the most severe acidification (pH 6.5), the overall expression of *rpl23a* remained relatively stable across the majority of experimental conditions (**Supplementary Table S1**). These results confirm that *rpl23a* is suitable as an internal reference gene for normalization in this study.

### 3.4 Expression of melanin synthesis genes is downregulated under acidified conditions

Transcript levels of melanin synthesis-related genes were strongly altered by seawater acidification. Both *tyr* and *per* displayed a broadly biphasic response profile, with transient transcriptional induction at early exposure followed by pronounced suppression during mid-development.

Under control conditions, *tyr* expression fluctuated moderately across the 9-day observation period (**Figure 3**). On Day 1, *tyr* transcript levels were significantly elevated in the pH 7.7 and pH 7.3 groups relative to controls (*p* < 0.05), with the strongest induction seen in the pH 7.3 treatment. No significant upregulation was detected in the pH 6.5 group at this stage. From Day 3 onward, *tyr* expression in the pH 7.3 and pH 6.5 groups decreased markedly and remained significantly lower than controls through most of the remaining experimental period (*p* < 0.05). A transient rebound in *tyr* expression occurred in the pH 7.3 group on Day 5, but this effect was not sustained. By Day 9, *tyr* expression in the pH 7.7 group increased sharply to exceed control levels (*p* < 0.05), while the pH 6.5 group retained significantly reduced expression at approximately 45% of the control value (*p* < 0.05).

**Figure 3:**
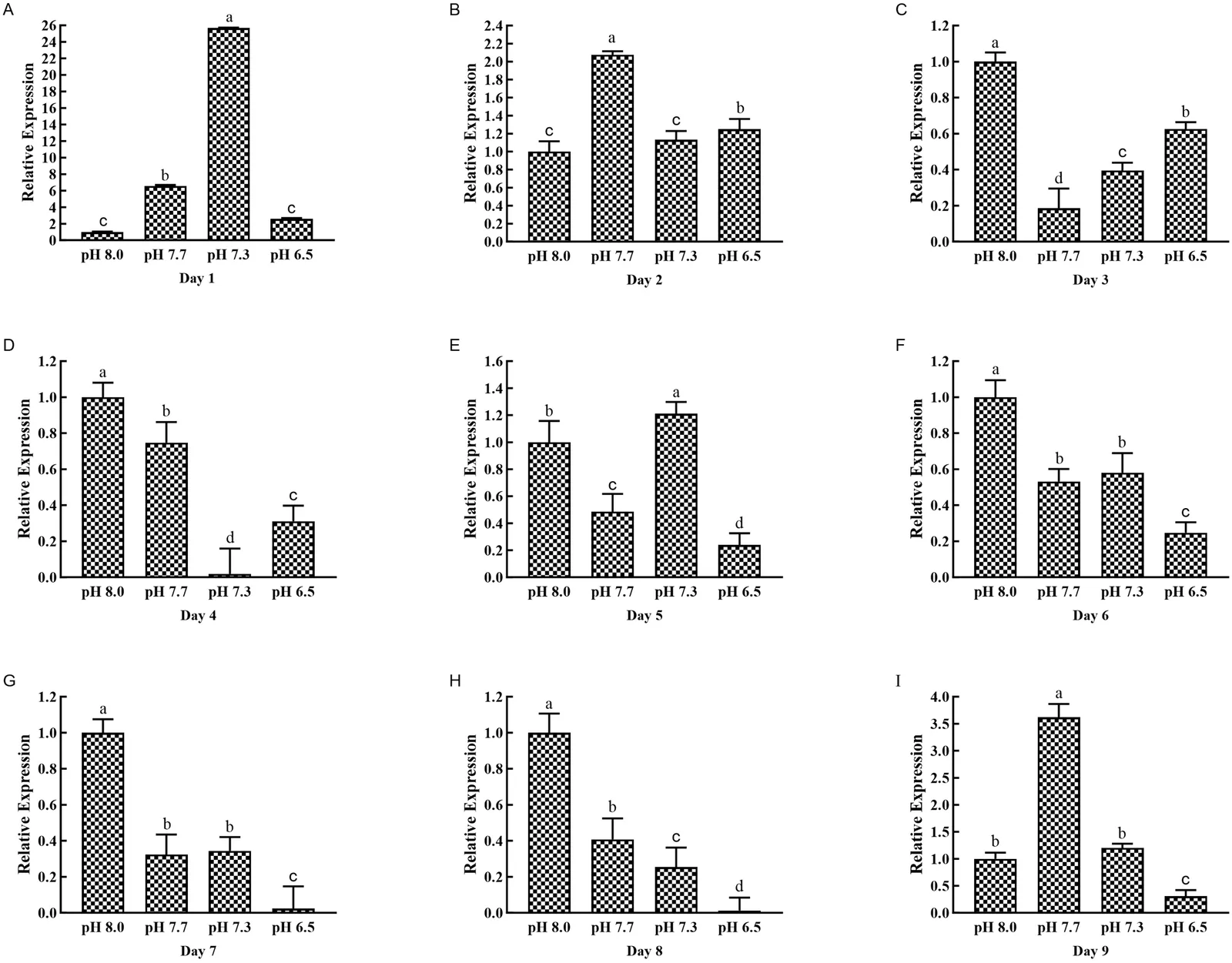
Relative expression of the tyrosinase (*tyr*) gene in the ink sac of *Sepia pharaonis* embryos. Panels **(A–I)** correspond to relative transcript levels of *tyr* from Day 1 to Day 9 under four different pH conditions (pH 8.0, 7.7, 7.3, and 6.5). Gene expression was quantified using qRT-PCR and normalized to the reference gene *rpl23a*. The expression level in the control group (pH 8.0) on Day 1 was set as the calibrator (value = 1). Bars represent the mean ± SD (*n* = 3 biological replicates). Different lowercase letters indicate significant differences between groups (P < 0.05); the same letter indicates no significant difference (P > 0.05).

The *per* gene showed a largely congruent expression pattern in response to acidification stress (**Figure 4**). On Day 1, all three acidified groups exhibited significantly higher *per* expression than controls (*p* < 0.01), with the pH 7.3 group showing the greatest magnitude of induction. Transcript levels declined thereafter across all acid treatments, with significant suppression observed at mid-developmental stages compared with controls (*p* < 0.05). In contrast to the sustained downregulation seen at pH 6.5, *per* expression in the pH 7.7 and pH 7.3 groups recovered progressively from Day 7 onward and rose significantly above control levels by Day 9 (*p* < 0.05). This coordinated transcriptional response aligns with the observed impairment of ink sac pigmentation and morphogenesis under acidified conditions.

**Figure 4:**
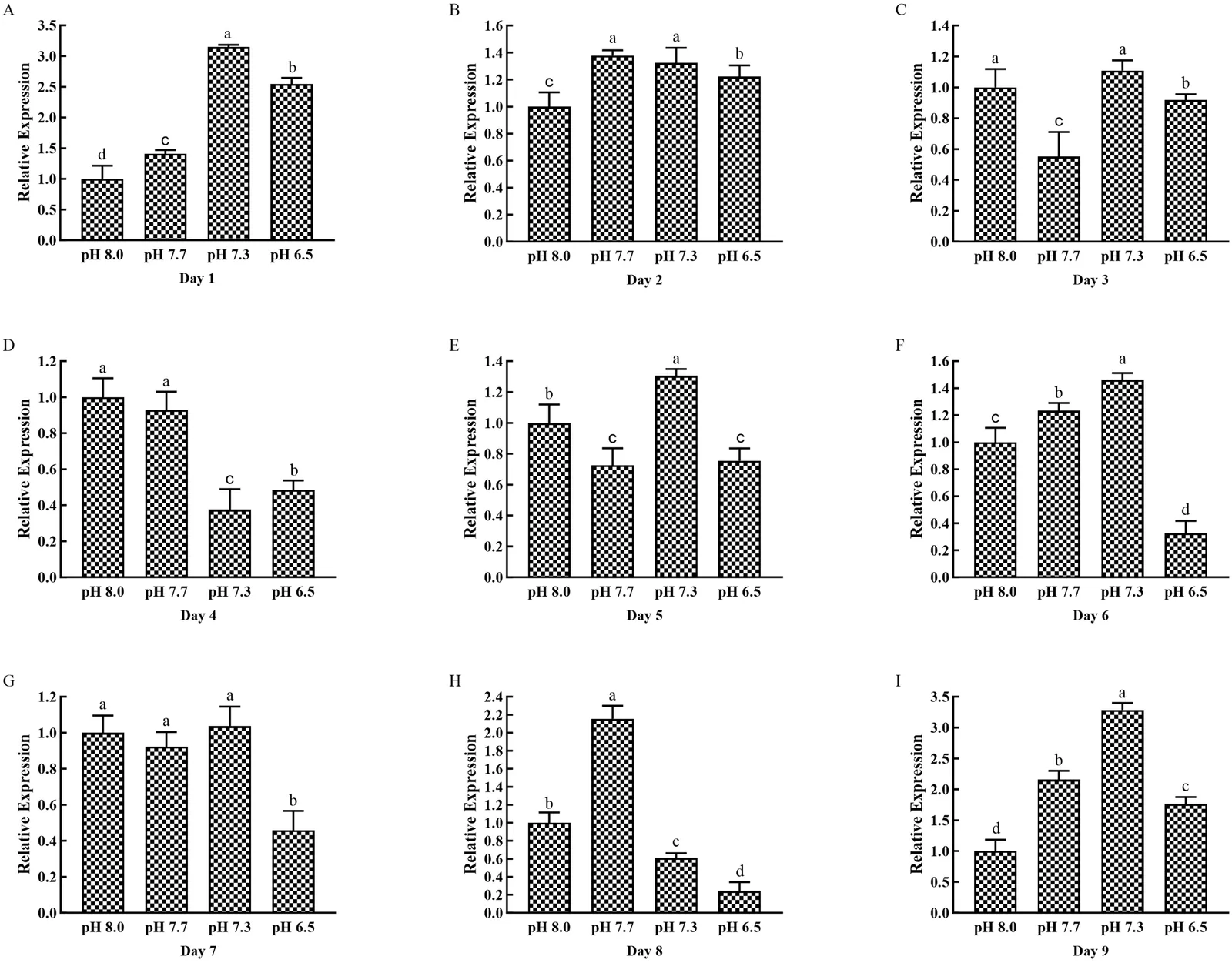
Relative expression of the peroxidase (*per*) gene in the ink sac of *Sepia pharaonis* embryos. Panels **(A–I)** correspond to relative transcript levels of *per* from Day 1 to Day 9 under four different pH conditions (pH 8.0, 7.7, 7.3, and 6.5). Gene expression was quantified using qRT-PCR and normalized to the reference gene *rpl23a*. The expression level in the control group (pH 8.0) on Day 1 was set as the calibrator (value = 1). Bars represent the mean ± SD (*n* = 3 biological replicates). Different lowercase letters indicate significant differences between groups (P < 0.05); the same letter indicates no significant difference (P > 0.05).

### 3.5 Expression of the regulatory gene spNOS is differentially modulated by acidification stress

The *spNOS* regulatory gene exhibited a temporal expression profile distinct from the core melanogenic enzymes *tyr* and *per* (**Figure 5**). Under control pH 8.0 conditions, *spNOS* transcript abundance fluctuated moderately across the developmental time course.

**Figure 5:**
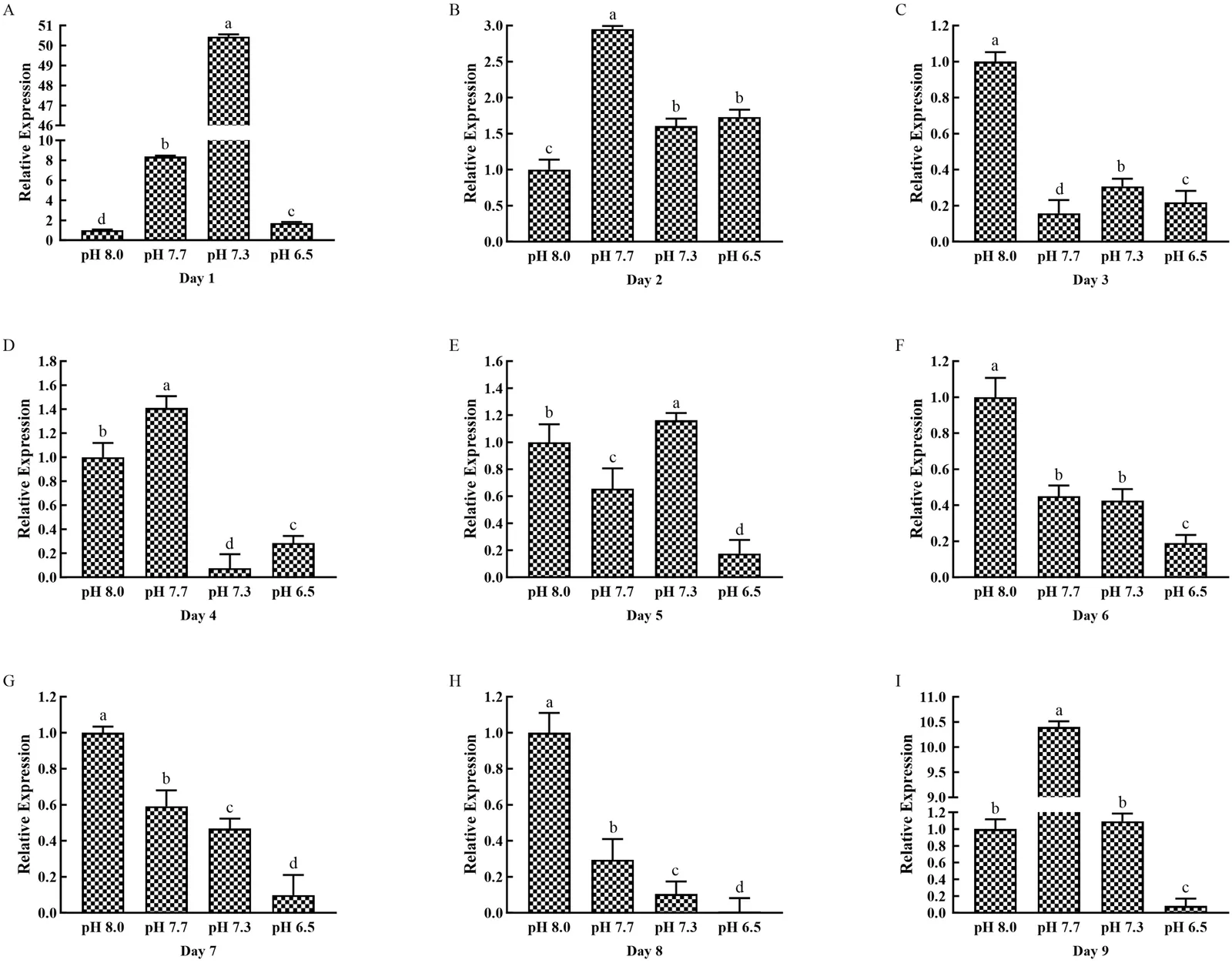
Relative expression of the nitric oxide synthase (*spNOS*) gene in the ink sac of *Sepia pharaonis* embryos. Panels **(A–I)** correspond to relative transcript levels of *spNOS* from Day 1 to Day 9 under four different pH conditions (pH 8.0, 7.7, 7.3, and 6.5). Gene expression was quantified using qRT-PCR and normalized to the reference gene *rpl23a*. The expression level in the control group (pH 8.0) on Day 1 was set as the calibrator (value = 1). Bars represent the mean ± SD (*n* = 3 biological replicates). Different lowercase letters indicate significant differences between groups (P < 0.05); the same letter indicates no significant difference (P > 0.05).

On Day 1, *spNOS* was significantly upregulated in all three acidified groups relative to controls (all *p* < 0.05), with the strongest induction seen at pH 7.3, consistent with an acute transcriptional stress response to initial acid exposure.

From Day 2 onward, expression trajectories diverged with acidification severity. Embryos at pH 6.5 showed progressive *spNOS* downregulation, reaching the lowest transcript levels on Day 8 (*p* < 0.01). Moderate acidification groups displayed fluctuating expression without a unidirectional trend; pH 7.3 transiently rose above control levels on Day 5 (*p* < 0.05) before declining again, while pH 7.7 remained moderately suppressed through most of this window.

By Day 9, *spNOS* expression remained significantly reduced in the pH 6.5 group (*p* < 0.05). In contrast, pH 7.7 showed pronounced upregulation to the peak value across all sampling points, significantly higher than controls (*p* < 0.05). No significant difference was observed between the pH 7.3 group and controls at this late stage.

Overall, only severe acidification induces sustained transcriptional repression of *spNOS*. Moderate acidification triggers a dynamic biphasic response, with late-stage upregulation limited exclusively to the pH 7.7 treatment.

### 3.6 Statistical control for salinity confounding

To evaluate whether the variation in salinity (23–26‰) among treatments confounded the effects of acidification, an ANCOVA was performed with salinity as a covariate. After controlling for salinity, the effects of pH on yolk sac area, mantle length, and the expression of *tyr*, *per*, and *spNOS* remained statistically significant (p < 0.05), indicating that pH was the primary driver of the observed biological responses.

## 4 Discussion

This study provides clear evidence that OA exerts pH-dependent detrimental effects on the embryonic development of *Sepia pharaonis*. Embryos reared under moderately acidified conditions (pH 7.7 and 7.3) completed development and hatching with delayed growth and reduced ink sac size, whereas severe acidification (pH 6.5) completely blocked normal development, causing yolk opacification and embryonic malformation. At the molecular level, severe acidification induced sustained downregulation of the melanin synthesis genes *tyr* and *per*, as well as the regulatory gene *spNOS*, while moderate acidification triggered a biphasic transcriptional response characterized by early suppression and late-stage rebound. It should be noted that the lower pH treatments (7.3 and 6.5), while exceeding most open-ocean projections, are representative of episodic acidification events in coastal and eutrophic systems, and thus provide insight into the physiological limits of embryonic tolerance under extreme environmental stress. Together, these findings demonstrate a consistent correspondence between phenotypic defects and molecular alterations, suggesting that OA is associated with alterations in both morphological and genetic processes underlying ink sac formation.

A potential molecular mechanism contributing to impaired ink sac development may involve the inhibition of melanogenesis. Tyrosinase catalyzes the rate-limiting step in melanin biosynthesis by oxidizing tyrosine to dopaquinone (Land et al., 2003), whereas peroxidase is involved in the final polymerization of melanin (Sarangarajan and Apte, 2006). In the present study, both genes were significantly downregulated under acidified conditions, particularly at pH 6.5, where *tyr* expression declined to only 45% of the control level by Day 9. This coordinated downregulation is consistent with the visibly reduced pigmentation in the mantle and ink sac of the embryos. A plausible explanation for this suppression is that decreased environment pH may interfere with intracellular acid-base balance, thereby affecting the enzymatic activity of tyrosinase and related proteins. Previous studies have suggested that external acidification can influence cellular pH regulation and enzyme function in aquatic organisms (Tresguerres and Hamilton, 2017), although this mechanism was not directly examined in the present study. Tyrosinase is known to be highly sensitive to pH, with maximal catalytic efficiency under neutral to slightly alkaline conditions (Song H. et al., 2021). Acidic stress may thus inhibit its activity (Chen et al., 2025), triggering negative feedback that reduces transcriptional expression of *tyr* and *per*. Such a mechanism has been observed in mollusks and fish exposed to acidified environments, where disrupted intracellular pH correlates with decreased melanin-related enzyme activity (Zhong and Wang, 2024; Chen et al., 2025). Notably, moderate acidification did not cause persistent transcriptional inhibition, and partial recovery of melanogenic gene expression was observed at late developmental stages, likely reflecting compensatory acclimation of embryonic acid-base regulation over time.

Beyond transcriptional suppression of melanogenesis-related genes, the observed developmental delay and organ underdevelopment can be interpreted within the framework of the energy trade-off hypothesis. Under acidified conditions, marine embryos expend additional energy maintaining acid-base balance, primarily through ATP-dependent ion transport mechanisms (Chille et al., 2022). This reallocation of metabolic energy may reduce the resources available for growth and differentiation of nonessential organs during embryogenesis. In *S. pharaonis*, the ink sac, although critical for post-hatching defense, is not essential for embryonic survival. Consequently, energy diverted toward pH homeostasis and basic metabolic maintenance could limit pigment cell proliferation and ink sac morphogenesis. Similar trade-offs have been reported in bivalve larvae and fish embryos under CO_2_-induced acidification, where increased ion-regulatory costs lead to reduced somatic growth and delayed organogenesis (Sganga et al., 2022). Therefore, the inhibited ink sac development observed here may reflect a systemic energy reallocation rather than organ-specific toxicity.

Unlike the core melanogenic enzymes, *spNOS* showed divergent sensitivity to acidification of different intensities. Nitric oxide synthase (NOS) produces NO, a signaling molecule crucial for the maturation of ink gland cells (Scheinker et al., 2005). Sustained downregulation of *spNOS* was observed exclusively under severe acidification (pH 6.5), suggesting a potential impairment of signaling pathways associated with developmental arrest at extremely low pH.signaling. Under moderate acidification, *spNOS* exhibited dynamic fluctuations rather than stable expression, with a prominent late-stage upregulation in the pH 7.7 group, suggesting activation of NO-mediated stress signaling during extended acid exposure.

Notably, *per* and *spNOS* showed universal transient upregulation across all acidified groups on Day 1, while *tyr* was only induced under moderate acidification. This pattern likely represents an acute stress response or compensatory regulation, wherein embryos initially attempt to counteract environmental change through transcriptional activation of key metabolic pathways. As acidification stress persisted, this early compensatory response failed in the severe acidification group, leading to sustained transcriptional suppression. In contrast, embryos under moderate acidification exhibited partial transcriptional recovery at later stages, indicative of partial acclimation to acidified conditions. Similar transient gene activation followed by acclimation or decline has been reported in other mollusks exposed to heavy metal or hypoxic stress, supporting the interpretation of an early-phase stress adaptation.

It is important to acknowledge a potential confounding factor in our gene expression analysis. Because the acidification treatments altered developmental rates, embryos from different pH groups sampled on the same chronological day may actually correspond to different developmental stages. Since the expression of melanogenesis-related genes (*tyr*, *per*, and *spNOS*) is tightly linked to the progression of organogenesis, the observed transcriptional differences between treatments could partially reflect the divergence in developmental stage rather than a direct pH effect. However, two lines of evidence suggest that pH still plays a significant role. First, the transient upregulation of *per* and *spNOS* on Day 1 occurred across all acidified groups when embryos were still at a comparable early developmental stage, indicating an acute transcriptional response to acid exposure independent of developmental progression. Second, the sustained suppression of *tyr* and *spNOS* in the pH 6.5 group persisted beyond the point of developmental arrest, where stage divergence alone cannot fully explain the continuously low transcript levels. While these findings suggest a direct association between pH stress and altered gene expression, we acknowledge that disentangling the effects of pH from those of developmental stage requires further investigation. Future studies should employ synchronized developmental staging (e.g., using morphological landmarks rather than chronological time) or single-embryo transcriptomics to isolate these effects.

Our results align partially with those of Wen et al. (2011), who reported that *S. pharaonis* juveniles exhibited high survival at pH 7.41 and 7.91 but experienced reduced pigmentation and lethargy at pH 5.91. The present findings extend this knowledge by identifying the embryonic stage as a critical window of sensitivity to acidification. Whereas juveniles can tolerate moderate pH reductions, embryos exhibit pronounced vulnerability, likely due to undeveloped regulatory mechanisms and limited buffering capacity.

Comparable developmental impairments have been documented in other cephalopods. For instance, Loligo vulgaris embryos show delayed organogenesis under acidified conditions, and Octopus vulgaris embryos experience altered gene expression related to shell and nervous system formation (Lacoue-Labarthe et al., 2011). Collectively, these results suggest that embryonic stages of cephalopods, despite lacking calcareous structures, are not immune to OA stress. Instead, acidification appears to target key developmental processes such as pigmentation, neural differentiation, and energy metabolism, functions crucial for post-hatching survival (Gunathilake et al., 2009; Lannig et al., 2010; Costa et al., 2022). Beyond cephalopods, similar dose-dependent developmental suppression has been observed in non-calcifying invertebrates like sea urchins and gastropods (Evans et al., 2013; Duquette et al., 2017; Leung et al., 2017), reinforcing the generality of our findings while highlighting the particular vulnerability of cephalopod embryos.

Ink production plays a central role in predator avoidance in newly hatched cuttlefish. The observed reduction in ink sac size and pigmentation under acidified conditions suggests that hatchlings may be less capable of deploying effective ink clouds for defense. This could lead to higher predation rates and reduced recruitment success in natural populations. Given the ecological importance and economic value of *S. pharaonis*, these findings imply that ongoing ocean acidification may threaten population stability and fishery sustainability. As embryonic sensitivity often determines reproductive success, impaired embryogenesis could have cascading effects on population dynamics, particularly in regions where coastal acidification intensifies due to eutrophication and anthropogenic CO_2_ emissions.

While this study provides valuable mechanistic insights, several limitations should be acknowledged. First, the experimental design used stable pH treatments, whereas natural seawater experiences diel and seasonal fluctuations. Second, only acidification was tested, while in reality, organisms face multiple concurrent stressors such as warming and hypoxia. Third, the analysis was restricted to three candidate genes; transcriptomic approaches would allow comprehensive mapping of the molecular networks affected by OA. In addition, replication at larger sample sizes and across developmental cohorts would enhance robustness. Fourth, our study only measured the mRNA abundance of three candidate genes. We did not directly quantify melanin content or tyrosinase/peroxidase/NOS enzyme activities. Therefore, the observed transcriptional changes suggest an association with, but cannot confirm direct functional causation of, impaired melanogenesis. Fifth, it should be noted that although *rpl23a* was stable under most conditions (overall CV = 8.27%), its expression variability slightly exceeded 10% on Day 9 under severe acidification (pH 6.5). This indicates that extreme stress may marginally affect its stability. Therefore, the gene expression results under severe acidification should be interpreted with caution, and future studies are recommended to validate multiple reference genes (e.g., using geNorm or NormFinder) to ensure robust normalization. Finally, two notable limitations regarding water chemistry and sampling design should be addressed. First, significant differences in salinity were detected among the treatment groups (p < 0.0001). Although ANCOVA confirmed that pH remained the primary driver after controlling for salinity, we cannot entirely exclude the possibility that the observed developmental and transcriptional responses are the result of combined effects of reduced pH and variable salinity. Future studies should implement stricter salinity control to isolate the specific effects of ocean acidification. Second, due to the destructive sampling design (5 embryos per tank were randomly sampled and dissected daily for gene expression analysis), we were unable to track the daily survival of individual embryos or calculate precise survival and hatching rates. Consequently, the survival and hatching outcomes are presented as qualitative descriptions rather than quantitative rates. Future studies should employ non-invasive tracking methods (e.g., time-lapse imaging) to quantify these endpoints more precisely.

Future research should investigate transgenerational effects, examining whether parental exposure to acidification can induce adaptive tolerance in offspring. The combined effects of acidification with other environmental stressors should also be explored to better reflect natural conditions. Future studies should incorporate enzymatic and biochemical assays (e.g., measuring tyrosinase activity and melanin content) to validate the functional consequences of these transcriptional changes. Integrating omics-based analyses with physiological and behavioral assays will be crucial for understanding how molecular changes translate into altered phenotypes and ecological outcomes. For instance, testing whether hatchlings with reduced ink sacs exhibit increased predation vulnerability would provide direct ecological validation of our findings.

## 5 Conclusions

In summary, ocean acidification disrupts the embryonic development of *S. pharaonis* through molecular suppression of melanogenesis. By connecting developmental phenotypes with underlying gene expression changes, this study offers a mechanistic framework for understanding how acidification compromises cephalopod resilience in future ocean environments.

## Data Availability

The original contributions presented in the study are included in the article/**Supplementary Material**. Further inquiries can be directed to the corresponding author.

## Supplementary material

Supplementary Figure 1 Effects of low pH on the embryonic development of *Sepia pharaonis*. Morphological images of embryos from Day 1 to Day 11 under control (pH 8.0) and acidified (pH 7.7, 7.3, and 6.5) conditions. Representative images illustrating the development of the ink sac from Day 1 to Day 11.

Supplementary Figure 2 Schematic diagram of the method for measuring mantle length (mm) in *Sepia pharaonis* embryos.

Supplementary Figure 3 Schematic diagram of the method for measuring yolk sac area (mm^2^) in *Sepia pharaonis* embryos.

Supplementary Figure 4 Effects of low pH on the morphogenesis of the ink sac in *Sepia pharaonis* embryos. The complete images illustrating the development of the ink sac from Day 1 to Day 11.

Supplementary Figure 5 Schematic diagram of the method for measuring ink sac area (mm^2^) in *Sepia pharaonis* embryos.
