# Supplementary material for: Ocean acidification impairs embryonic development and disrupts ink sac gene expression in the pharaoh cuttlefish (Sepia pharaonis)
Source: Front Physiol. 2026 Oct 23;17:1979863. doi: 10.3389/fphys.2026.1979863 (PMC13739555; doi:10.3389/fphys.2026.1979863)
Supplement: Supplementary file 1 [file Table2.docx]

**Table S2. Changes in mantle length (mm) of *Sepia pharaonis* embryos under different pH treatments over time.**

| **Time**（Day） | **pH 6.5** | **pH 7.3** | **pH 7.7** | **pH 8.0** |
| --- | --- | --- | --- | --- |
| Day 1 | 0.724 ± 0.023 ^c^ | 1.000 ± 0.015 ^a^ | 0.872 ± 0.006 ^b^ | 0.661 ± 0.005 ^d^ |
| Day 2 | 1.139 ± 0.002 ^a^ | 0.813 ± 0.011 ^c^ | 0.880 ± 0.012 ^b^ | 0.767 ± 0.016 ^d^ |
| Day 3 | 0.654 ± 0.017 ^c^ | 0.775 ± 0.018 ^b^ | 0.780 ± 0.004 ^b^ | 0.886 ± 0.006 ^a^ |
| Day 4 | 0.804 ± 0.005 ^d^ | 0.837 ± 0.009 ^c^ | 0.991 ± 0.012 ^b^ | 1.128 ± 0.011 ^a^ |
| Day 5 | 0.887 ± 0.012 ^d^ | 0.980 ± 0.014 ^c^ | 1.330 ± 0.018 ^a^ | 1.207 ± 0.013 ^b^ |
| Day 6 | 1.096 ± 0.020 ^b^ | 1.375 ± 0.006 ^a^ | 1.485 ± 0.024 ^a^ | 1.483 ± 0.031 ^a^ |
| Day 7 | 1.107 ± 0.019 ^b^ | 1.472 ± 0.003 ^a^ | 1.486 ± 0.006 ^a^ | 1.505 ± 0.023 ^a^ |
| Day 8 | 0.852 ± 0.020 ^d^ | 1.318 ± 0.017 ^c^ | 1.995 ± 0.025 ^a^ | 1.765 ± 0.013 ^b^ |
| Day 9 | 0.835 ± 0.010 ^d^ | 1.318 ± 0.003 ^c^ | 1.649 ± 0.021 ^b^ | 1.758 ± 0.022 ^a^ |
| Day 10 | - | 1.334 ± 0.007 ^b^ | - | 1.632 ± 0.008 ^a^ |
| Day 11 | - | 1.259 ± 0.016 ^b^ | - | 1.889 ± 0.021 ^a^ |

**Note: Data are presented as mean ± standard deviation (SD) (n = 3 replicate tanks). Different lowercase letters within the same row indicate significant differences among pH treatments at that specific time point (Two-way ANOVA followed by Tukey's post-hoc test, p < 0.05). "-" indicates that data collection was terminated for that group (pH 6.5 and pH 7.7 groups were terminated on Day 9 due to 100% mortality and failure to hatch, respectively).**
