# Supplementary material for: Ocean acidification impairs embryonic development and disrupts ink sac gene expression in the pharaoh cuttlefish (Sepia pharaonis)
Source: Front Physiol. 2026 Oct 23;17:1979863. doi: 10.3389/fphys.2026.1979863 (PMC13739555; doi:10.3389/fphys.2026.1979863)
Supplement: Supplementary file 2 [file Table3.docx]

**Table S3. Changes in yolk sac area (mm²) of *Sepia pharaonis* embryos under different pH treatments over time.**

| **Time**（Day） | **pH 6.5** | **pH 7.3** | **pH 7.7** | **pH 8.0** |
| --- | --- | --- | --- | --- |
| Day 1 | 1.859 ± 0.016 ^c^ | 1.339 ± 0.006 ^d^ | 2.552 ± 0.019 ^b^ | 2.787 ± 0.004 ^a^ |
| Day 2 | 1.265 ± 0.005 ^d^ | 2.209 ± 0.005 ^b^ | 1.607 ± 0.013 ^c^ | 2.384 ± 0.025 ^a^ |
| Day 3 | 1.567 ± 0.012 ^c^ | 1.654 ± 0.013 ^b^ | 1.627 ± 0.009 ^b^ | 1.691 ± 0.011 ^a^ |
| Day 4 | 1.991 ± 0.017 ^b^ | 2.079 ± 0.013 ^a^ | 1.687 ± 0.007 ^c^ | 1.395 ± 0.005 ^d^ |
| Day 5 | 2.058 ± 0.010 ^a^ | 1.466 ± 0.005 ^c^ | 1.322 ± 0.016 ^d^ | 1.552 ± 0.005 ^b^ |
| Day 6 | 1.924 ± 0.011 ^a^ | 1.601 ± 0.006 ^b^ | 1.272 ± 0.014 ^d^ | 1.378 ± 0.007 ^c^ |
| Day 7 | 2.065 ± 0.021 ^a^ | 1.590 ± 0.005 ^b^ | 1.202 ± 0.005 ^c^ | 1.097 ± 0.021 ^d^ |
| Day 8 | 1.614 ± 0.012 ^a^ | 1.352 ± 0.009 ^b^ | 1.073 ± 0.005 ^c^ | 0.812 ± 0.006 ^d^ |
| Day 9 | 2.313 ± 0.010 ^a^ | 1.628 ± 0.009 ^b^ | 1.051 ± 0.009 ^c^ | 0.695 ± 0.005 ^d^ |
| Day 10 | - | 1.251 ± 0.014 ^a^ | - | 0.704 ± 0.013 ^b^ |
| Day 11 | - | 1.131 ± 0.009 ^a^ | - | 0.555 ± 0.001 ^b^ |

**Note: Data are presented as mean ± standard deviation (SD) (n = 3 replicate tanks). Different lowercase letters within the same row indicate significant differences among pH treatments at that specific time point (Two-way ANOVA followed by Tukey's post-hoc test, p < 0.05). "-" indicates that data collection was terminated for that group (pH 6.5 and pH 7.7 groups were terminated on Day 9 due to 100% mortality and failure to hatch, respectively).**
