# Supplementary material for: Ocean acidification impairs embryonic development and disrupts ink sac gene expression in the pharaoh cuttlefish (Sepia pharaonis)
Source: Front Physiol. 2026 Oct 23;17:1979863. doi: 10.3389/fphys.2026.1979863 (PMC13739555; doi:10.3389/fphys.2026.1979863)
Supplement: Supplementary file 3 [file Table4.docx]

**Table S4. Changes in ink sac area (mm²) of *Sepia pharaonis* embryos under different pH treatments over time.**

| **Time**（Day） | **pH 6.5** | **pH 7.3** | **pH 7.7** | **pH 8.0** |
| --- | --- | --- | --- | --- |
| Day 1 | 0.003 ± 0.001 ^c^ | 0.012 ± 0.001 ^a^ | 0.007 ± 0.001 ^b^ | 0.005 ± 0.000 ^bc^ |
| Day 2 | 0.011 ± 0.001 ^a^ | 0.003 ± 0.000 ^c^ | 0.006 ± 0.001 ^b^ | 0.007 ± 0.001 ^b^ |
| Day 3 | 0.012 ± 0.001 ^a^ | 0.004 ± 0.001 ^c^ | 0.006 ± 0.001 ^b^ | 0.004 ± 0.000 ^c^ |
| Day 4 | 0.014 ± 0.001 ^a^ | 0.006 ± 0.001 ^b^ | 0.013 ± 0.001 ^a^ | 0.007 ± 0.001 ^b^ |
| Day 5 | 0.011 ± 0.001 ^b^ | 0.005 ± 0.001 ^c^ | 0.019 ± 0.001 ^a^ | 0.012 ± 0.001 ^b^ |
| Day 6 | 0.005 ± 0.000 ^c^ | 0.016 ± 0.001 ^b^ | 0.032 ± 0.003 ^a^ | 0.013 ± 0.001 ^b^ |
| Day 7 | 0.008 ± 0.001 ^c^ | 0.019 ± 0.001 ^b^ | 0.032 ± 0.001 ^a^ | 0.033 ± 0.001 ^a^ |
| Day 8 | 0.015 ± 0.001 ^d^ | 0.019 ± 0.001 ^c^ | 0.041 ± 0.001 ^b^ | 0.044 ± 0.001 ^a^ |
| Day 9 | 0.016 ± 0.002 ^d^ | 0.035 ± 0.003 ^c^ | 0.045 ± 0.002 ^b^ | 0.064 ± 0.001 ^a^ |
| Day 10 | - | 0.049 ± 0.002 ^b^ | - | 0.068 ± 0.003 ^a^ |
| Day 11 | - | 0.057 ± 0.001 ^b^ | - | 0.081 ± 0.004 ^a^ |

**Note: Data are presented as mean ± standard deviation (SD) (n = 3 replicate tanks). Different lowercase letters within the same row indicate significant differences among pH treatments at that specific time point (Two-way ANOVA followed by Tukey's post-hoc test, p < 0.05). "-" indicates that data collection was terminated for that group (pH 6.5 and pH 7.7 groups were terminated on Day 9 due to 100% mortality and failure to hatch, respectively).**
